# Supplementary material for: A set of multi-entry identification keys to African frugivorous flies (Diptera, Tephritidae)
Source: Zookeys. 2014 Jul 24;(428):97–108. doi: 10.3897/zookeys.428.7366 (PMC4143993; doi:10.3897/zookeys.428.7366)
Supplement: Supplementary material 6 — Key to Ceratitis [file zookeys-428-097-s006.zip › SF6_ZooKeys_key to Ceratitis/key/SF6_ZooKeys_key to Ceratitis/Media/Html/Ceratitis faceta .htm]

Ceratitis (Pterandrus) faceta Enderlein


***Ceratitis*** **(*Pterandrus*) *faceta* Enderlein**

*Ceratitis* *faceta* Enderlein, 1920: 349.

Body
length: 4.57 (4.45-4.70) mm; wing length: 4.80 (4.70-4.90) mm.

Male

Head: Antenna yellowish orange. First flagellomere three
times as long as pedicel. Arista with short rays; basoventral rays shorter and
sparser than basodorsal rays. Frons convex; yellow; with short scattered
setulae largely of same color as frons. Frontal setae well developed. Face
white. Genal seta and setulae black, latter poorly developed. Occiput mainly
dark, ventral margin only pale.

Thorax:
Postpronotal lobe dark yellow, large black spot occupying most of the
postpronotal lobe so that it appears to have largely the same color as scutum. Mesonotum:
shiny black, with dark pilosity; no streaks or spots, sutural lines more
brownish. Scapular setae dark. Scutellum shiny black; basally near lateral
margins with paler spot. Anepisternum on ventral half brown, with conspicuous
silvery shine when viewed from front; pilosity pale except ventral to
anepisternal seta where few setulae dark.

Legs:
Largely dark; coxa, trochanter, femur, basal 0.33 of foretibia and hindtibia
and whole of midtibia black, otherwise yellow; setation typical for subgenus,
mixed pale and dark. Foreleg: femur without bush posteroventrally, only few
dispersed dark setulae. Midleg: femur anteriorly with yellow oblique line on
distal 0.33, silvery when viewed from certain angle; ventrally silvery when
viewed from certain angle, on distal half with black setulae; tibia moderately
broadened, with black feathering along dorsal 0.9 and ventral 0.66; anteriorly
with second row of short black setulae along median half. Hindleg: femur at
apical 0.33 with longer black setulae dorsally and ventrally; forming
relatively dense feathering ventrally.

Wing:
Marginal band forming continuous band with anterior part of discal band;
cubital band free; medial band absent; crossvein R-M opposite middle of discal
cell. Apex of vein R_1_ distal to level of crossvein R-M. Crossvein
DM-Cu oblique anterobasally.

Abdomen:
Shiny black. Tergites 2 and 4 with silvery band along respectively posterior
half and 0.66. Setation and banding typical for subgenus. (Male terminalia not
dissected)

Female

As
in male except for the following characters. Antenna orange. Frons darker colored,
orange. Genal setulae well developed. Anepisternum with dark pilosity on
ventral part, without conspicuous silvery shine. DM-Cu crossvein straight. Legs
without feathering; yellow, tibia darkened on basal part, femora dark brown;
forefemur posteroventrally with dark pilosity. Ovipositor shorter than
preabdomen; Aculeus at least 10 times longer than wide; tip pointed and lateral
margin slightly concave.

(Description
after De Meyer & Freidberg, 2006)
